# Detection of risk for type 2 diabetes and its relationship with metabolic alterations in nurses*

**DOI:** 10.1590/1518-8345.3002.3161

**Published:** 2019-07-18

**Authors:** Bernarda Sánchez-Jiménez, Gabriela Chico-Barba, Ana Lilia Rodríguez-Ventura, Reyna Sámano, Daniela Veruete-Bedolla, Rosa María Morales-Hernández

**Affiliations:** 1 Instituto Nacional de Perinatología, Subdirección de Investigación en Intervenciones Comunitarias, Ciudad de México, México.; 2 Instituto Nacional de Perinatología, Departamento de Nutrición y Bioprogramación, Ciudad de México, México.; 3 Universidad Panamericana, Facultad de Ciencias de la Salud, Escuela de Enfermería, Ciudad de México, México.; 4 Universidad del Valle de México, Licenciatura en Nutrición, Ciudad de México, México.

**Keywords:** Risk Factors, Metabolic Diseases, Diabetes Mellitus Type 2, Nurses, Lipids, Blood Glucose, Fatores de Risco, Doenças Metabólicas, Diabetes Mellitus Tipo 2, Enfermeiras e Enfermeiros, Lipídeos, Glicemia, Factores de Riesgo, Enfermedades Metabólicas, Diabetes Mellitus Tipo 2, Enfermeros, Lípidos, Glicemia

## Abstract

**Objective:**

to detect the risk of development of type 2 diabetes in nurses and its relationship with metabolic alterations.

**Method:**

cross-sectional study, with 155 nurses. The variables investigated were: sociodemographic, body mass index, waist circumference, waist-hip index, lipid profile, basal glycemia and oral glucose tolerance curve. The *Finnish Diabetes Risk Score* was used to collect data.

**Results:**

155 nurses were included, with an average age of 44 years and 85% were overweight or obese. 52% had a family history of diabetes and 21% had occasional hyperglycemia. With respect to the risk, 59% were identified with moderate and very high risk for type 2 diabetes*.* Glucose, insulin, glycosylated hemoglobin A1c and insulin resistance increased in parallel to the increased risk for type 2 diabetes, although lipids did not increase. 27% of the sample had impaired fasting glycemia. 15% had glucose intolerance and 5% had type 2 diabetes.

**Conclusion:**

there was a high detection rate of people at risk for type 2 diabetes (59%) and the high and very high risk score was associated with high levels of glycosylated hemoglobin A1c, glucose, insulin and insulin resistance, but not with lipids.

## Abstract (pt)

**Objetivo:**

identificar o risco de desenvolvimento de diabetes tipo 2 em enfermeiras e sua relação com as alterações metabólicas.

**Método:**

estudo transversal, com 155 enfermeiras. As variáveis investigadas foram: sociodemográficas, índice de massa corporal, a circunferência da cintura, índice cintura-quadril, perfil lipídico, a glicemia basal e a curva oral de tolerância à glicose. Para a coleta de dados utilizou-se o *Finnish Diabetes Risk* Score.

**Resultados:**

Das 155 (100%) enfermeiras, a média de idade foi de 44 anos e 85% apresentavam sobrepeso ou obesidade; 52% tinham história familiar de diabetes e 21%, hiperglicemia ocasional. Em relação ao risco, 59% foram identificados com risco moderado e muito alto de diabetes tipo 2*.* A glicose, a insulina, a hemoglobina glicosilada A1c e a resistência à insulina aumentaram paralelamente ao aumento do risco de diabetes tipo 2, embora os lipídios não tenham aumentado. 27% das participantes apresentaram glicemia em jejum alterada, 15%, intolerância à glicose e 5%, diabetes tipo 2.

**Conclusão:**

houve uma elevada taxa de detecção de risco de diabetes tipo 2 (59%) e a pontuação de risco alto e muito alto foi associado com níveis elevados de hemoglobina glicosilada A1c, glicose, insulina e resistência à insulina, mas não com lipídios.

## Abstract (es)

**Objetivo:**

identificar el riesgo de desarrollo de diabetes tipo 2 en enfermeras y su relación con alteraciones metabólicas.

**Método:**

estudio transversal, con 155 enfermeras. Las variables investigadas fueron: sociodemográficas, el índice de masa corporal, circunferencia de cintura, índice cintura-cadera, perfil de lípidos, glucemia basal y curva de tolerancia oral a la glucosa. Para la recolección de datos se utilizó el *Finnish Diabetes Risk Score*.

**Resultados:**

De las 155 enfermeras, la edad promedio fue 44 años y 85% tenía sobrepeso u obesidad. El 52% tenía antecedentes familiares de diabetes de primera línea, el 21% hiperglucemia ocasional. Con relación al riesgo, se identificaron 59% con riesgo de diabetes tipo 2 moderado y muy alto*.* Glucosa, insulina, hemoglobina glucosa A1c y la resistencia a la insulina incrementaron paralelos al aumento del riesgo de diabetes tipo 2, aunque los lípidos no. El 27% de las enfermeras presentó glucemia basal alterada. El 15% tuvo intolerancia a la glucosa y 5% diabetes tipo 2.

**Conclusión:**

la detección de riesgo de diabetes tipo 2 fue elevada (59%) y el puntaje de riesgo alto y muy alto se relacionó con valores mayores de hemoglobina glucosa A1c, glucosa, insulina y resistencia a la insulina pero no con lípidos.

## Introduction

Chronic non-communicable diseases have become a worldwide epidemic that threatens life expectancy and quality of life and increases cases of death and disability^(^ ^1^ ^)^. Type 2 Diabetes mellitus (T2DM) is becoming one of the most prevalent diseases in the 21st century and is a global public health challenge^(^ ^2^ ^)^. The World Health Organization (WHO) estimated in 2014 that 422 million people had diabetes, of which 90% had T2DM^(^ ^3^ ^)^. According to the International Diabetes Federation, China, India, United States, Brazil and Mexico are, in this order, the countries with the highest number of individuals suffering from diabetes^(^ ^4^ ^)^. Some of the risk factors for developing T2DM are genetic and environmental. In this regard, there are cohort studies that show the importance of nutrition and lifestyle in the development of diabetes in health professionals and nurses, and over 90% of cases were potentially preventable^(^ ^5^ ^)^. In Mexico, the prevalence of diabetes in the general population is 9.4%^(^ ^6^ ^)^. Although there is a slight increase in this prevalence in relation to previous years, health surveillance and prevention of complications are very far from being achieved. The American Diabetes Association (ADA), recommends testing for this disease through fasting glycemia and, if necessary, oral glucose tolerance curve in asymptomatic adults with overweight or obesity^(^ ^7^ ^)^. Hence, the early detection of diabetes and its risk factors can affect the appearance of its complications, which affect the quality of life of people and the costs of medical care. A quick, simple and self-applicable tool is the Finnish Diabetes Risk Score (FINDRISC) questionnaire, which is used to assess the risk of developing diabetes in the following 10 years^(^ ^8^ ^)^.

T2DM is a chronic degenerative disease prevalent in the general population, and health workers are not excluded from presenting this type of disease. In the case of the nursing staff, their lifestyle, in addition to the long working hours, different shifts, stress and anxiety that they face daily, makes it difficult the adoption of healthy habits^(^ ^9^ ^–^ ^11^ ^)^ and could lead them to a higher risk of developing diabetes than other members of the health staff. In addition, these professionals, from the epidemiological point of view, are considered as a vulnerable group because of the risk to their physical and emotional health^(^ ^9^ ^)^.

The interest in identifying nurses at high risk of developing diabetes lies in the influence they exert on the population to motivate them to take care of their health, so it is crucial that they first take care of their own health by identifying their risk for the disease. For this reason, the objective of this study was to identify the risk of development of type 2 diabetes in nurses and its relationship with metabolic alterations.

In addition, to our knowledge, there have been no studies associating the results of a non-invasive technique for detecting T2DM with clinical, anthropometric and biochemical variables in this population. Therefore, these results support the development of health promotion strategies aimed at the nursing staff, as the effect would be like a “mirror”. That is, on the one hand, to strengthen their knowledge and motivate them for personal care and, on the other hand, to ensure that they have the necessary tools to promote health and give guidance to the population.

## Method

Cross-sectional analytical study carried out from April 2016 to May 2017 in the nursing staff of an institution specialized in reproductive health in Mexico City. This study was based on the WHO ethical principles (Declaration of Helsinki) and was approved by the Institutional Research, Ethical and Biosafety Committees (registration number: 212250-3300-11402-01-15). Participants were recruited by means of personal invitation and posters, and their participation took place after signing an informed consent form, in which the objectives and procedures of the study were mentioned, as well as the risks, benefits and confidentiality of the data. The sample was sequential and intentional, consisted of 158 participants, of which three were men and due to this small number, they were not included in the statistical analyzes, and 155 nurses composed the sample. The inclusion criteria were that they had a base contract of all shifts, services and categories in the institution. Nursing professionals with a previous diagnosis of diabetes and pregnant women were excluded.

By means of a questionnaire, all the participants were asked about their academic training, type of patients they cared for as nurses, length of service and sociodemographic characteristics. The FINDRISC questionnaire was applied, which is a tool that has shown a sensitivity of 81% and a specificity of 76% to predict the development of diabetes through the use of noninvasive clinical variables^(^ ^8^ ^)^. It was designed by the Finnish National Diabetes Programme in 2001, validated by the National Public Institute of Helsinki^(^ ^12^ ^–^ ^13^ ^)^, and in several countries such as Spain, among others^(^ ^14^ ^)^. This instrument allows to estimate the individual's risk of developing T2DM and to classify him into one of the five risk groups. The most accurate cut-off point for predicting a high risk of developing diabetes (≥20% in 10 years) is 15 or more points^(^ ^15^ ^)^.

The questionnaire comprises eight variables: 1) body mass index, 2) waist circumference, 3) physical activity, 4) consumption of fruits and vegetables, 5) age, 6) use of hypertensive, 7) high blood glucose and 8) family history of diabetes. This instrument was validated for use in Spanish^(^ ^16^ ^)^ and has already been used in other studies in Mexico^(^ ^17^ ^–^ ^19^ ^)^.

After completing the questionnaires and a previous 12-hour fasting, a blood sample was collected to measure the levels of glucose and insulin, glycosylated hemoglobin A1c (HbA1c), total cholesterol, low density lipoprotein (LDL-cholesterol), high-density lipoprotein (HDL-cholesterol) and triglycerides. For those participants included in the “*very high risk*” category (equal to or greater than 15 points) according to FINDRISC, or with HbA1c levels greater than 5.7%, a 2-hour oral glucose tolerance curve (OGTC) was performed, with their prior consent, to corroborate the diagnosis. Biochemical determinations were carried out in the Nutrition and Bioprogramming laboratory in the same institution. In the anthropometric assessment, body weight and height were determined in order to calculate the body mass index (BMI) and classify it according to the WHO criteria. Waist and hip circumferences were also measured. The anthropometry was performed according to Lohman's techniques. Blood pressure was measured using a mercury sphygmomanometer, after a five-minute rest and according to international standards.

The following classification criteria were used for glucose levels: a) *Without Diabetes*, when fasting plasma glucose was <100 mg/dL and/or 2-hour blood glucose <140mg/dL; b) *Pre Diabetes*, fasting plasma glucose in the 100–125 mg/dL range, and/or 2-hour blood glucose in the 140–199 mg/dL range, and c) *Diabetes*, when fasting plasma glucose ≥126 mg/ dL, or 2-hour blood glucose ≥200 mg/dL. HbA1c was also considered for prediabetes diagnosis when the levels were in the 5.7%-6.4% range, and for diabetes ≥6.5%, as recommended by the ADA's Standards of Medical Care in Diabetes^(^ ^7^ ^)^.

For the statistical analysis, the type of distribution of the quantitative variables was determined by Kolmogorov-Smirnov test, and considering as a normal distribution when p>0.05. The averages were calculated with standard deviations (mean ± standard deviation) and median with an interquartile range (25^th^ percentile - 75^th^ percentile) for continuous variables, depending on their distribution. The frequencies and percentages were obtained from the categorical variables. The Pearson's Chi-square test (X^2^) was used to analyze the risk category and sociodemographic data. The Kruskal-Wallis test was used to identify the differences between the biochemical measures and the group of risk for T2DM. For all the analyzes, a value of p<0.05 was considered significant.

## Results

In total, 155 nurses were evaluated, with an average age of 44 years (±8.45), average length of service of 21 years (±.9.08); 60% (n=94) of the participants cared for severe patients, and of these, 59% (n=55) worked in the morning shift. 42% had a university degree. 85% of participants were overweight or obese, and the average waist size was 88 cm (±.11.83). 52% had a family history of diabetes; 21% had high blood glucose detected at some time, and 14% had a diagnosis of high blood pressure and/or treatment. When analyzing the healthy habits, it was identified that 25% of participants performed physical activity and 43% consumed vegetables and fruits in their daily diet. 27% of the population had impaired fasting glucose. The OGTC test was performed in 88 cases and alterations were found in 20%; 15% had glucose intolerance (prediabetes) and 5% had T2DM. Regarding the estimated risk of the FINDRISC, 92 (59%) participants with *moderate* to *very high risk* were identified. 59% of participants who were in the high risk category had prediabetes, based on fasting glucose and HbA1c.

The general characteristics of the participants according to the FINDRISC categories are shown in Table 1. Of the 74 (48%) participants aged 45 years or older, 30 (41%) were at *high*/*very high* risk for diabetes, a similar situation was observed in 24 (44%) nurses who had studied in technical schools, which was not statistically significant in both cases. In relation to the risk for diabetes according to marital status, it did not matter if they were married or single, since the percentages were similar in the *slightly elevated* risk category in both (p=0.256). It was observed that those who cared for outpatients and/or did not have direct contact with patients, the risk for T2DM was *high* (47%) when compared to those who care for serious patients (22%). Regarding the length of professional experience, it was observed that the greater the number of years worked, the greater the risk for T2DM.

**Table 1:** General characteristics of the nurses according to category of risk for type 2 diabetes, based on FINDRISC*. Mexico City, Mexico, 2016-2017

Variable		Risk for Type 2 Diabetes					
		Low	Slightly elevated	Moderate	High	Very high	p- Value
		(n=12)	(n=51)	(n=41)	(n=44)	(n=7)	
Age (years)†							
	<45	9(11)	28(35)	23(28)	18(22)	3(4)	0.249
	≥45	3 (4)	23 (31)	18(24)	26(35)	4(5)	
Schooling†							
	Technical (General)	2(4)	14(26)	14(26)	23(42)	1(2)	0.121
	University degree	5(8)	23(35)	18(28)	14(21)	5(8)	
	Master's/doctorate	5(14)	14(39)	9(25)	7(19)	1(3)	
Marital status†							
	Married	4(4)	33(34)	28(29)	27(28)	5(5)	
	Single	8(14)	18(31)	13(23)	17(29)	2(3)	0.256
Shift†							
	Morning	9(9)	30(30)	28(29)	28(29)	3(3)	0.736
	Evening	1(7)	5(35)	4(29)	4(29)	0	
	Night	2(5)	16(37)	9(21)	12(28)	4(9)	
Type of Patient†							
	Serious	8(9)	34(36)	28(30)	21(22)	3(3)	0.080
	Non-serious	3(12)	6(24)	7(28)	6(24)	3(12)	
	Outpatient and without contact with patient	1(3)	11(30)	6(17)	17(47)	1(3)	
Years of Professional Experience‡		16.5(5.2-26)	21(10-26)	24(18.5-26)	25(19.5-29)	28(15-31)	0.103

* FINDRISC = Finnish Diabetes Risk Score;

† Data expressed as frequency (%), Pearson's Chi-squared test;

‡ Data expressed as median (p25-p75), Kruskal-Wallis test

Table 2 shows that the body mass index, waist circumference and waist/hip ratio increase as the risk category for T2DM increases (p<0.001).

**Table 2:** Clinical characteristics of the nurses according to the risk category for type 2 diabetes, based on FINDRISC*. Mexico City, Mexico, 2016-2017

Variable	Risk for Type 2 Diabetes					
	Low	Slightly elevated	Moderate	High	Very high	p-Value
	(n=12)	(n=51)	(n=41)	(n=44)	(n=7)	
BMI (kg/m^2^)†	23.8	26.5	28.2	29.8	33.1	<0.01
	(22.9-26.0)	(25-28.6)	(26.1-31.2)	(27.1-35.6)	(28.1-36.5)	
Waist (cm)†	78	85.4	89.1	92.2	99	<0.01
	(75.1-79.5)	(80.5-90)	(83.2-95)	(87.1-100)	(87-101)	
Waist-Hip Ratio†	0.81	0.85	0.86	0.87	0.87	<0.01
	(0.76-0.86)	(0.82-0.88)	(0.81-0.89)	(0.83-0.90)	(0.85-0.92)	

* FINDRISC = Finnish Diabetes Risk Score;

† Data expressed as median (p25-p75), Kruskal-Wallis test

Table 3 shows that the biochemical parameters such as glucose, insulin and HbA1c, increased their values directly as the risk for diabetes increased in the FINDRISC test. In contrast, total cholesterol, HDL cholesterol and LDL cholesterol did not exhibit the same behavior. However, their values were higher in the *very high risk* category compared to the values of the *low risk* category. Regarding triglycerides, it was possible to observe that the highest value (165 mg/dL) was present in the *high risk* group and shown to be statistically significant (p<0.01).

**Table 3:** Biochemical profile of the nurses according to risk category for type 2 diabetes, based on FINDRISC*. Mexico City, Mexico, 2016-2017

Variable	Risk for Type 2 Diabetes					
	Low	Slightly elevated	Moderate	High	Very high	p-Value
	(n=12)	(n=51)	(n=41)	(n=44)	(n=7)	
Fasting glycemia	92.6	92	92	99.2	106	<0.01
(mg/dL)†	(85.0-96.8)	(86.7-97.3)	(85-97.2)	(91.7-107)	(96-109)	
Insulin	5.6	10.1	11	16.4	19	<0.01
(I.U.)†	(4.6-6.7)	(6.6-15.7)	(7.3-18.9)	(12-27)	(12-25)	
HbA1c	5.5	5.5	5.6	5.7	6	0.018
(%)†	(5.3-5.8)	(5.4-5.7)	(5.4-5.8)	(5.5-6)	(5.5-6.1)	
HOMA	1.3	2.4	2.3	4.2	4.5	0.001
Index‡	(1-1.5)	(1.4-3.6)	(1.7-4.4)	(3-7.2)	(3-6.6)	
Triglycerides	121.5	114	112	165	147	<0.01
(mg/dL)†	(93-144.2)	(94-182)	(101.5-164)	(131-208.2)	(124-171)	
Total Cholesterol	193	193	187	186	222	0.044
(mg/dL)†	(167.7-208.2)	(163-210)	(170.5-204)	(175.2-206.7)	(203-293)	
HDL Cholesterol	51.8	46.7	46.3	42.8	52.2	0.017
(mg/dL)†	(46.8-63.3)	(41.5-58)	(42.8-52.8)	(39.8-49.8)	(45-63)	
LDL Cholesterol	107.5	117.8	108.2	108.2	142	0.022
(mg/dL)†	(96-122.3)	(93.7-128.6)	(100.4-122)	(100.2-126.4)	(137-202.1)	

* FINDRISC = Finnish Diabetes Risk Score;

† Data expressed as median (p25-p75), Comparison between medians by Kruskal-Wallis;

‡ HOMA Index = insulin resistance index (Homeostatic Model Assessment)

## Discussion

Diabetes is a major public health problem in the country, both due to its complications and its consequences, including mortality. In this study, a 15% frequency of glucose intolerance (prediabetes) was observed, which is lower than that reported in other countries, including Mexico, where it varies from 19.9 to 43.2%^(^ ^17^ ^–^ ^18^ ^,^ ^20^ ^–^ ^21^ ^)^ in similar samples and in the general population, but higher than the frequency of 6.7% found in Ecuador^(^ ^22^ ^)^. The frequency found in this study is higher than that reported in 2018, which mentions that about 7.5% of adults in Mexico have prediabetes^(^ ^23^ ^)^. Based on these findings, solutions could be sought to minimize the alterations observed in these health workers who are at great risk of developing diabetes in the next 10 years, in order to delay the progression of the disease and avoid cardiovascular complications^(^ ^14^ ^)^. In addition, it should be remembered that prediabetes increases the absolute risk for T2DM in the short term by 3 to 10 times^(^ ^24^ ^)^. Regarding body mass index, the percentage of overweight or obesity observed was high (85%), even higher than the 31% reported in Cuba^(^ ^25^ ^)^, 64% in Ecuador^(^ ^22^ ^)^, and 72.5% reported in Mexico at a national level^(^ ^6^ ^)^. It is known that this factor can be harmful to health. A study conducted in New Zealand with adults, found that the prevalence of prediabetes was 32.2% in obese patients and 26.9% in those who were overweight^(^ ^26^ ^)^.

On the other hand, there are lifestyle intervention studies that have shown benefits, such as the Finnish Diabetes Prevention Programme, which has achieved lifestyle changes and reduced the incidence of diabetes^(^ ^27^ ^)^. In addition, the Diabetes Prevention Programme reduced the incidence of diabetes by 58% when compared to the 31% who have used metformin^(^ ^28^ ^)^. Both were intervention studies, with an average length of three years and included non-diabetic men and women with impaired glucose. As part of a healthy lifestyle, one must perform physical exercises, which is crucial to maintaining good physical and mental health. For decades, it has been shown that little physical activity is associated with an increase in the rates of ischemic heart disease, various types of cancer, obesity, type 2 diabetes, high blood pressure, dyslipidemia, among others, besides increasing the early mortality rate and overall mortality rate. According to a systematic review and meta-analysis^(^ ^29^ ^)^, in which the dose-response of physical activity in the above-mentioned diseases was assessed, it was observed that those who performed more physical activity than recommended, had a 14% reduction in the risk for breast cancer, 21% for colon cancer, 28% for diabetes, 25% for ischemic heart disease and 26% for ischemic stroke. In the present study, it was identified that 75% of participants did not perform physical activity, a value higher than that reported in the general population in Cuba (34%)^(^ ^25^ ^)^ and also higher than reported in a group of nurses in Australia (54%)^(^ ^30^ ^)^. It is important to mention that the populations of these countries are different from those of Mexico, where there is no culture of prevention. In a study conducted in Ecuador, an increase in prediabetes was found in the health staff who did not perform sufficient of physical activity (5.6%), compared to 1.1% of those who performed it^(^ ^22^ ^)^. In this regard, some cohort studies have provided convincing epidemiological evidence that physical activity and a healthy diet would prevent most cases of T2DM^(^ ^5^ ^)^. Regarding nutrition, in this study, less than half of the participants (43%) consumed vegetables and fruits in their daily diet, a lower percentage (71%) than that reported by Ortíz-Contreras^(^ ^18^ ^)^, but higher than that found in Cuba (29%)^(^ ^25^ ^)^. A review of 21 articles on nutrition in nursing professionals found that social, organizational, physical factors and the country are determinants for the healthy eating habits in nurses at the workplace^(^ ^31^ ^)^.

When analyzing the waist circumference (WC), 127 (82%) participants were found with altered WC, i.e., greater than or equal to 80 cm, a circumference greater than that found for health professionals in Ecuador (63%)^(^ ^22^ ^)^. The average WC in our study was 88.9 cm, which is lower than that reported for the rural population (97.2 cm) in Guadalajara, Mexico^(^ ^21^ ^)^. It is worth mentioning that the results found in this study are not comparable with others, as the case of Cuba, because they consider as altered a WC ≥88cm. In addition, as expected, we observed that in this study, WC increased as the risk estimation also increased.

Regarding the lipid profile, it is worth mentioning that it was expected that the higher the category of risk for T2DM, the higher the values of triglycerides, total cholesterol and LDL-cholesterol, and the lower the values of HDL-cholesterol. However, there is no linear trend between categories and, the FINDRISC might not be able to identify well the individuals with high risk for T2DM based on their serum lipids, at least in this population.

We believe that the cut-off point we have used, equal to or greater than 15 points (high/very high risk), was appropriate to identify people at high risk of developing prediabetes and T2DM. However, it is necessary to perform other tests to achieve improved diagnosis, such as the fasting plasma glucose test and the postprandial plasma glucose test, as recommended by some authors^(^ ^14^ ^)^.

Our study highlights the health problem presented by the nursing workers, since more than half of the participants exhibited moderate or high risk of developing diabetes, which was associated with metabolic alterations. The risk of having T2DM or some other chronic noncommunicable disease is latent and constant, and the use of easy and quick tools for their detection, such as the FINDRISC questionnaire, can help in the prevention and awareness of self-care. The use of this questionnaire is useful to identify individuals at risk of developing prediabetes and/or detect T2DM and other metabolic alterations early, as well as to develop and implement strategies aimed at reducing this high risk.

The present study has several limitations. The nursing staff of the institute showed little interest in participating, only 33% accepted. There are different reasons for this: it is likely that individuals who already had a previous diagnosis of a chronic noncommunicable disease did not want to participate for fear of being exposed to their workmates. In addition, there was little support from some service managers to allow their staff to attend to evaluations, possibly due to excessive workload, which is common among health workers. Another explanation could be that the nursing staff gives less importance to their own health, when it should be more relevant. Another limitation is that it is not reasonable to make comparisons by sex, since the vast majority is women, so it would be interesting to include male nurses in order to identify differences in the factors of risk for T2DM. Due to the aforementioned, these results cannot be extrapolated to the nursing workers in the country and should be considered with caution. In addition, the sample was not representative. In view of the above, if the sample size was increased, it is possible that the tendency and significance of the association between the risk of developing T2DM and the serum lipid values would be as expected. It is worth mentioning that due to the design of the study, the associations found cannot be considered as causality.

## Conclusion

The detection of moderate to very high risk of developing T2DM was high (59%) and the high and very high risk score was associated with high levels of HbA1c, glucose, insulin and insulin resistance, but this association was not observed with the lipid profile. Besides the biochemical and clinical variables, there are labor characteristics associated with a higher detection rate of people at risk of developing T2DM.
